# Human agonistic TRAIL receptor antibodies Mapatumumab and Lexatumumab induce apoptosis in malignant mesothelioma and act synergistically with cisplatin

**DOI:** 10.1186/1476-4598-6-66

**Published:** 2007-10-22

**Authors:** Larisa L Belyanskaya, Thomas M Marti, Sally Hopkins-Donaldson, Stefanie Kurtz, Emanuela Felley-Bosco, Rolf A Stahel

**Affiliations:** 1 Laboratory of Molecular Oncology, Clinic and Policlinic of Oncology, University Hospital of Zürich, Häldeliweg 4, 8044 Zürich, Switzerland; 2 Materials-Biology Interactions Laboratory, Materials Science and Technology (Empa), Lerchenfeldstrasse 5, 9014 St. Gallen, Switzerland

## Abstract

**Background:**

The incidence of malignant pleural mesothelioma (MPM) is associated with exposure to asbestos, and projections suggest that the yearly number of deaths in Western Europe due to MPM will increase until 2020. Despite progress in chemo- and in multimodality therapy, MPM remains a disease with a poor prognosis. Inducing apoptosis by tumor necrosis factor-related apoptosis-inducing ligand (TRAIL) or agonistic monoclonal antibodies which target TRAIL-receptor 1 (TRAIL-R1) or TRAIL-R2 has been thought to be a promising cancer therapy.

**Results:**

We have compared the sensitivity of 13 MPM cell lines or primary cultures to TRAIL and two fully human agonistic monoclonal antibodies directed to TRAIL-R1 (Mapatumumab) and TRAIL-R2 (Lexatumumab) and examined sensitization of the MPM cell lines to cisplatin-induced by the TRAIL-receptor antibodies. We found that sensitivity of MPM cells to TRAIL, Mapatumumab and Lexatumumab varies largely and is independent of TRAIL-receptor expression. TRAIL-R2 contributes more than TRAIL-R1 to death-receptor mediated apoptosis in MPM cells that express both receptors. The combination of cisplatin with Mapatumumab or Lexatumumab synergistically inhibited the cell growth and enhanced apoptotic death. Furthermore, pre-treatment with cisplatin followed by Mapatumumab or Lexatumumab resulted in significant higher cytotoxic effects as compared to the reverse sequence. Combination-induced cell growth inhibition was significantly abrogated by pre-treatment of the cells with the antioxidant N-acetylcysteine.

**Conclusion:**

Our results suggest that the sequential administration of cisplatin followed by Mapatumumab or Lexatumumab deserves investigation in the treatment of patients with MPM.

## Background

Malignant pleural mesothelioma (MPM) is a generally fatal thoracic neoplasia that arises from the pleural lining. In the majority of the patients, a history of occupational exposure to asbestos can be elicited [1]. Taking into account a latency period of 20–50 years and a decline in workplace exposure to asbestos in Europe since the 1970s, it is estimated that the number of men dying from MPM in Europe will double each year until a peak is reached in about between 2015 and 2020 [2,3].

No chemotherapy regimen for mesothelioma has proven curative, although several treatments are valuable for palliation. The clinically best documented chemotherapy is a combination of cisplatin with an antifolate. A large phase III study comparing the combination of cisplatin and pemetrexed with cisplatin alone demonstrated a superior response, survival and a better quality of life for the combination [4,5]. For earlier stages of disease, specialized centers offer multimodality therapy with adjuvant or neoadjuvant chemotherapy, radical surgery with or without radiotherapy [6]. However, despite such aggressive treatment most patients have disease recurrence within 2 years. Therefore, new therapeutic options are needed for more effective treatment of this malignancy. As demonstrated by our *in vitro *investigations, the combination of cisplatin-based chemotherapy with agonistic TRAIL receptor antibodies might be a promising option.

Tumor necrosis factor (TNF)-related apoptosis-inducing ligand (TRAIL) is a type II transmembrane protein belonging to the TNF family of death ligands. Four TRAIL receptors have been identified of which two, TRAIL-R1/DR4 and TRAIL-R2/DR5, are capable of transducing an apoptotic signal whereas the other two receptors (TRAIL-R3/DcR1, TRAIL-R4/DcR2) act as antagonists since they lack death domains and thus cannot engage the apoptotic machinery [7,8]. An additional receptor, osteoprotegrin, has been identified but its activity is still matter of debate because of its low affinity for TRAIL at 37°C [9]. TRAIL can preferentially induce apoptosis in a variety of tumor cell types, whereas normal cells do not appear to be sensitive [10]. This property suggests TRAIL-R targeting is an excellent strategy for selective cancer therapy and oncology trials with TRAIL and TRAIL-R human agonistic antibodies have been initiated [11,12].

Apoptosis-inducing mechanisms by human agonistic TRAIL-R antibodies Mapatumumab and Lexatumumab are thought to be similar to TRAIL-mediated apoptosis [13]. TRAIL-induced cell death is triggered by the interaction of the ligand with TRAIL-R1 or TRAIL-R2 to assemble the death-inducing signaling complex. The latter forms when death receptor ligation triggers association of the intracellular adaptor, Fas-associated death domain (FADD) with the cytoplasmic tail of the receptor. FADD then recruits procaspase-8, which undergoes spontaneous autoactivation. Activated caspase-8, in turn, cleaves and activates the effector caspases-3, -6 and -7 which cleave cellular substrates to execute cell death [7,8]. Recent data suggest the existence of considerable cross-talk between the extrinsic and intrinsic death signalling pathways. Caspase-8, a key player of this communication platform, can proteolytically activate the BH3 only family member Bid, which induces Bax- and Bak-mediated release of cytochrome c and Smac/DIABLO from mitochondria [14]. Resistance to TRAIL can occur by different mechanisms, including lack of TRAIL apoptosis receptors, death receptor mutations [15], and enhanced expression of TRAIL-decoy receptors [16]. FLIP, which bears structural similarity to caspase-8, but lacks caspase-8 activity, can inhibit death receptor-mediated signalling by binding to FADD [17]. Both forms of FLIP, the long form c-FLIP_L _and the short form c-FLIP_S _can compete for apical caspase recruitment to the DISC, whereas FLIP_L _can also inhibit the full processing of caspase-8 [18].

MPM cells have been found by others to be resistant or to have a low susceptibility to TRAIL-induced apoptosis, and require either FLIP_L _siRNA, chemotherapeutic drugs, α-tocopheryl succinate or cycloheximide to be combined with TRAIL for apoptosis to occur [19-22]. However, these studies were performed with a small number of established human MPM cell lines only and it remains unknown whether the majority of MPM cell lines and primary cultures are indeed resistant to TRAIL combined with chemotherapy. In addition, no information exists on the sensitivity of MPM cells to two fully human agonistic monoclonal antibodies which target TRAIL-R1 (Mapatumumab) and TRAIL-R2 (Lexatumumab) although they have the advantage over TRAIL of a longer plasma half-life and a higher specificity [23].

In the present study, we compared the sensitivity of 13 MPM cell lines or primary cultures to TRAIL and to two fully human agonistic monoclonal antibodies which target TRAIL-R1 and TRAIL-R2, and examined the apoptosis sensitization of the MPM cell lines with different sensitivity to Mapatumumab or Lexatumumab by the cytotoxic drug cisplatin.

## Results

### Expression of TRAIL receptors in MPM cell lines or primary cultures

The expression of the four membrane-bound TRAIL-receptors was analyzed in a large panel of commercially available and well-established MPM cells lines obtained from human biopsies or pleural effusions [24,25]. Flow cytometry analyses performed on non-permeabilized cells with monoclonal antibodies raised against the extracellular domains of TRAIL-R1 and -R2, respectively, demonstrated expression of both receptors at the cell surface of MPM cells, with higher TRAIL-R2 than TRAIL-R1 expression in the majority of cells analysed (Table 1). In contrast, the two decoy receptors TRAIL-R3 and -R4 were either not detected or expressed at the very low levels at the cell surface of all cell lines (Table 1).

**Table 1:** Evaluation of TRAIL receptors cell surface expression in MPM cell lines. Expression of TRAIL-R1, TRAIL-R2, TRAIL-R3 and TRAIL-R4 was evaluated by flow cytometry. The values represent the fluorescence intensity of the receptors normalized for the negative control. Data are expressed as mean ± s.d. from three independent experiments. *n.e.-not expressed

	TRAIL-receptors			
MPM cell lines	TRAIL-R1	TRAIL-R2	TRAIL-R3	TRAIL-R4

*TRAIL-resistant*				
H2052	14.0 ± 0.4	58.0 ± 3.1	2.6 ± 0.1	1.7 ± 0.4
SPC212	30.5 ± 0.2	64.0 ± 2.4	1.2 ± 0.1	4.2 ± 0.8
ZL34	10.9 ± 0.5	65.5 ± 4.2	1.5 ± 0.2	2.0 ± 0.5
H226	12.7 ± 0.8	66.6 ± 6.5	n.e*	n.e
*TRAIL-partially resistant*				
SDM6	15.8 ± 0.3	33.6 ± 3.2	1.3 ± 0.1	n.e
SDM13	10.3 ± 0.2	15.3 ± 0.4	1.2 ± 0.3	1.4 ± 0.2
*TRAIL-sensitive*				
H2452	1.5 ± 0.1	34.0 ± 2.0	2.5 ± 0.2	1.7 ± 0.3
SCP111	33.0 ± 1.0	33.6 ± 1.5	2.1 ± 0.2	1.5 ± 0.2
ZL5	13.2 ± 0.2	55.7 ± 3.2	1.1 ± 0.1	1.3 ± 0.2
ZL55	32.8 ± 2.0	38.2 ± 3.5	n.e	1.2 ± 0.4
H28	26.4 ± 0.4	22.0 ± 0.8	1.3 ± 0.2	2.5 ± 0.5
SDM4	24.5 ± 0.3	58.0 ± 4.2	1.4 ± 0.2	1.5 ± 0.1
MSTO-211H	2.3 ± 0.1	60.4 ± 3.2	1.3 ± 0.3	1.3 ± 0.2

### Activity of TRAIL in MPM cell lines or primary cultures

**Figure 1:**
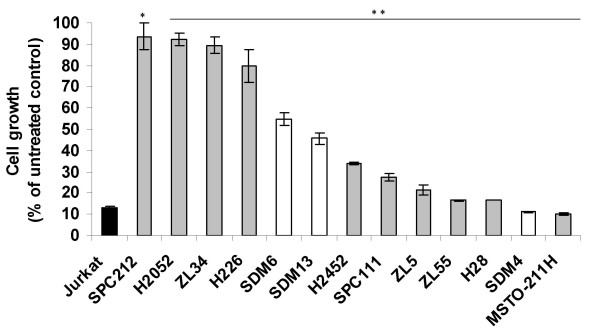
**Activity of TRAIL in MPM cell lines or primary cultures**. Jurkat T cell leukemic cells (black column), commercially available and/or well-established MPM cell lines (grey columns) and primary MPM cultures (white columns) were incubated with 100 ng/ml His-TRAIL, and cell proliferation was determined by MTT assay 72 h thereafter. Absorbance values obtained with untreated cells maintained under identical conditions were taken as 100%. Data represent means of three independent experiments and bars indicate standard deviations. **, *P *< 0.01 and *, *P *< 0.05 compared to untreated cells.

We have explored the biological effect of recombinant human TRAIL on MPM cells. For this purpose, 13 MPM cells lines were treated with 100 ng/ml His-TRAIL for 72 h, and cell viability was measured. Jurkat T cell leukemic cells are sensitive to TRAIL-mediated cell killing [26] and were used as a positive control. A significant inhibitory effect of TRAIL could be observed in all 13 tested cell lines (Figure 1). In detail, seven (MSTO-211H, SDM4, H28, ZL55, ZL5, SPC111, H2452,) were sensitive (defined as >60% cell death), two (SDM13, SDM6) were partially resistant (defined as 20–60% cell death) and the remaining four (H2052, SPC212, ZL34 and H226) were resistant (defined as < 20% cell death) to TRAIL. These data demonstrate that sensitivity of MPM cells to TRAIL is very heterogeneous. There was no correlation between the level of TRAIL death receptor expression and sensitivity to TRAIL.

### Activity of agonistic fully human anti-TRAIL death receptor monoclonal antibodies Mapatumumab and Lexatumumab in MPM cell lines or primary cultures

**Figure 2:**
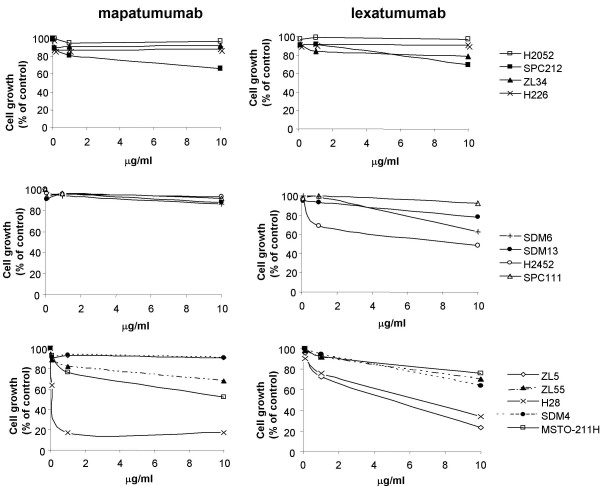
**Activity of agonistic fully human anti-TRAIL death receptor monoclonal antibodies Mapatumumab and Lexatumumab in MPM cell lines or primary cultures**. MPM cell lines or primary cultures were treated with 0.01 μg/ml, 1 μg/ml and 10 μg/ml Mapatumumab or Lexatumumab, and cell proliferation was determined by MTT assay 72 h thereafter. Absorbance values obtained with untreated cells maintained under identical conditions were taken as 100%. Representative data of three independent experiments showing similar results are shown here.

TRAIL-R1 and -R2 may be differentially involved in transmitting apoptotic signals [10,27,28] and the relative contribution of each death receptor to apoptosis induction in MPM cells expressing both receptors is unknown. To investigate the relative contribution of TRAIL-R1 and -R2 in apoptosis induction, MPM cell lines and primary cultures were incubated with increasing concentrations of specific human selective agonistic monoclonal antibodies for 72 h. Six out of thirteen MPM cell lines were more sensitive to Lexatumumab than to Mapatumumab (SDM13, ZL34, SDM6, SDM4, H2452, ZL5) and only two out of thirteen were more sensitive to Mapatumumab (MSTO-211H, H28) (Figure 2 and Table 2). Two cell lines showed similar sensitivity to Mapatumumab and Lexatumumab (ZL55, SPC111) and almost complete resistance to both antibodies (10 μg/ml) was detected in three cell lines (H2052, SPC212, H226). As observed for TRAIL, no correlation between the level of receptor expression (Table 1) and the sensitivity to antibodies could be found.

**Table 2:** Reduction in cell viability of MPM cells after incubation with Mapatumumab or Lexatumumab monoclonal antibodies. The percentage of reduction in cell viability was measured after incubation with 10 μg/ml Mapatumumab (Mapa) or Lexatumumab)Lexa) monoclonal antibodies. Data are expressed as mean ± s.d. from five independent experiments. **, *P *< 0.01 and *, *P *< 0.05 compared to untreated cells

	Reduction in cell viability (%) induced by	
	
MPM cell lines	10 μg/ml Mapa	10 μg/ml Lexa
*Mapa and Lexa resistant (< 20% cell death)*		
H2052	3 ± 11	2 ± 9
SPC212	9 ± 5 *	7 ± 8
H226	5 ± 11	4 ± 6
*more sensitive to Lexa*		
ZL5	9 ± 3 **	77+11 **
H2452	7 ± 3 **	52 ± 7 **
SDM4	9 ± 9	36 ± 9 **
SDM6	14 ± 14	37 ± 14 *
ZL34	8 ± 5 *	21 ± 10 **
SDM13	13 ± 3 *	22 ± 1 **
*more sensitive to Mapa*		
H28	83 ± 2 **	66 ± 20 *
MSTO-211H	48 ± 6 **	24 ± 11 *
*with similar sensitivity to Mapa and Lexa*		
SPC111	33 ± 14 **	31 ± 11 **
ZL55	31 ± 6 **	30 ± 9 **

### Cisplatin sensitizes MPM cells to Mapatumumab- or Lexatumumab- mediated cytotoxicity

**Figure 3:**
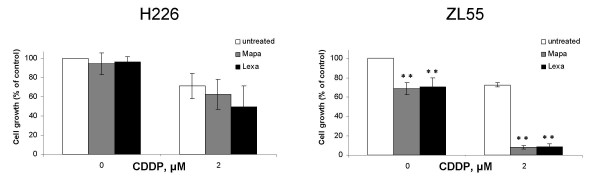
**Cisplatin sensitizes MPM cells to Mapatumumab- or Lexatumumab- mediated cytotoxicity**. Human agonistic TRAIL receptor antibodies Mapatumumab (Mapa) and Lexatumumab (Lexa) enhance the activity of cisplatin in MPM cells lines. H226 and ZL55 cells were cultured with or without 2 μM cisplatin for 24 h. Cells were subsequently left untreated or treated with 10 μg/ml Mapatumumab or Lexatumumab and cell proliferation was determined 72 h later as described under experimental procedures. Absorbance values obtained with untreated cells maintained under identical conditions were taken as 100%. Data represent means of three independent experiments and bars indicate standard deviations. **, *P *< 0.01 and *, *P *< 0.05 compared to cells not treated with anti-TRAIL receptor antibodies.

Several studies have shown that chemotherapeutic agents and radiotherapy sensitize tumor cells to Mapatumumab or Lexatumumab [29]. Therefore we tested how pre-treatment with cisplatin affects the response to agonist-TRAIL receptor antibodies in the MPM cell lines H226 and ZL55, which are agonist-TRAIL receptor antibodies resistant and sensitive, respectively. Incubation of H226 cell line with 2 μM cisplatin alone resulted in 29% cell death and addition of either Mapatumumab or Lexatumumab 24 h later (both at 10 μg/ml) did not significantly increase dead cell fraction (Fig. 3). However, a synergistic effect of cisplatin and agonist TRAIL receptor antibodies was observed in ZL55 cell line (Fig. 3).

**Figure 4:**
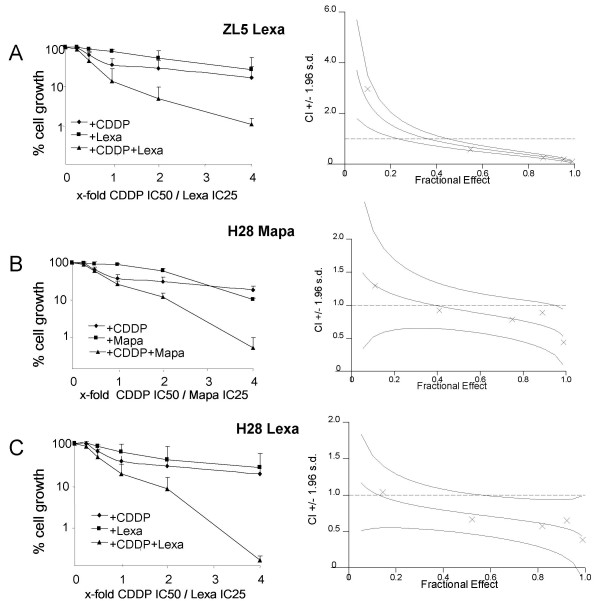
**Synergy of concurrent treatment with anti-TRAIL receptor antibodies and cisplatin**. Concurrent treatment with Lexatumumab (Lexa) monoclonal antibody enhances the activity of cisplatin in ZL5 MPM cells. ZL5 MPM cells were treated with single treatments or combinations of 2.12 μM cisplatin and/or 1.01 μg/ml Lexatumumab or fractions/multiples of those concentrations at a constant ratio from 0.25- to 4-fold. Responses were determined by MTT assay after 72 h and bars indicate standard deviations. Synergy was quantified by Combination Index (CI) analysis and expressed as CI versus fraction affected. By this method, CI<1 indicates synergy; CI = 1 indicates an additive effect; and CI>1 indicates antagonism. 96% confidence intervals are shown on CI plots. Data shown represent mean values from three independent experiments. ***B ***and ***C***. Concurrent treatment with Mapatumumab (Mapa) or Lexatumumab monoclonal antibodies enhance the cytotoxicity of cisplatin in H28 cells. Experiments were performed as described under *A*, except that cisplatin, Mapatumumab and Lexatumumab were used at a concentration of 6.61 μM, 0.13 μg/ml and 0.40 μg/ml, respectively, and fractions/multiples thereof. Data represent means of three independent experiments and bars indicate standard deviations.

To further characterize cisplatin-induced sensitization, we compared the effects of concurrent treatment with cisplatin and agonist TRAIL receptor antibodies in the cell lines ZL5 (sensitive only to Lexatumumab) and H28 (sensitive to both Mapatumumab and Lexatumumab). The synergy was evaluated in ZL5 cells exposed to single treatment or combinations of 2.12 μM cisplatin (IC50) and/or 1.01 μg/ml Lexatumumab (IC25) or fractions/multiples of those concentrations at a constant ratio from 0.25- to 4-fold (Fig. 4). Synergy was quantified by Combination Index (CI) analysis and expressed as CI versus fraction affected. By this method, CI<1 indicates synergy; CI = 1 indicates an additive effect; and CI>1 indicates antagonism. 96% confidence intervals are shown on CI plots. Low doses (0.25 and 0.5 times cisplatin IC50) of combined treatment with cisplatin and Lexatumumab resulted in decreased cell growth of ZL5 cells compared to single agent-treatments although no synergistic effect was detectable. Higher doses (1–4 times cisplatin IC50) of cisplatin and Lexatumumab reduced cell growth synergistically compared to either agent alone, with CI values lower than 0.6 (Fig. 4).

When the same strategy was applied to H28 cells, reduced cell growth was observed after treatment with low doses (0.25 times cisplatin IC50) of cisplatin in combination with Mapatumumab or Lexatumumab compared to single agent treatments but no synergism was detectable. However, higher doses (0.5–4 times cisplatin IC50) of cisplatin in combination with Mapatumumab or Lexatumumab synergistically inhibited cell growth, with CI values decreasing from 0.9 to 04 and from 0.7 to 0.4 for cisplatin in combination with Mapatumumab or Lexatumumab, respectively (Fig. 4).

Taken together, these results indicate that both, cisplatin pre- or concurrent treatment, are able to modulate the response induced by anti-TRAIL receptor antibodies Mapatumumab/Lexatumumab.

### Post- but not pre-treatment with agonist TRAIL receptor antibodies enhances cisplatin cytotoxicity in MPM cells

**Figure 5:**
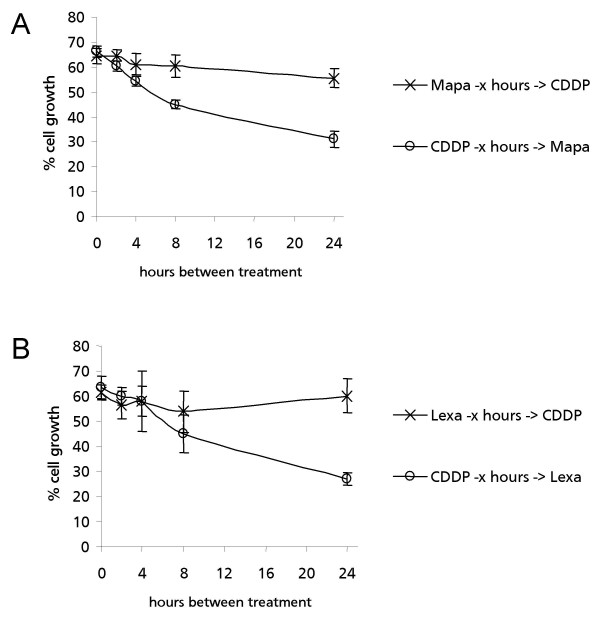
**Effect of pre- and post-treatment with either Mapatumumab or Lexatumumab on cisplatin cytotoxicity**. **A**. Effects of pre- and post-treatment with Mapatumumab (Mapa) on cisplatin cytotoxicity in H28 cells. (X) H28 cells were pre-treated with 0.1 μg/ml Mapatumumab and incubated for various times (0–24 h) followed by treatment with 4.0 μM cisplatin and responses were determined by MTT assay after 48 h. (o) H28 cells were treated with 4.0 μM cisplatin and post-treated with 0.1 μg/ml Mapatumumab after various times (0–24 h). Responses were determined by MTT assay after 48 h. ***B***. Effect of pre- and post-treatment with Lexatumumab (Lexa) on cisplatin cytotoxicity in H28 cells. Experiments were performed as described in *A*, except that Lexatumumab at a concentration of 0.4 μg/ml was used instead of Mapatumumab. Absorbance values obtained with untreated cells maintained under identical conditions were taken as 100%. Data represent means of three independent experiments and bars indicate standard deviations.

We examined whether the synergistic interaction between agonist TRAIL receptor antibodies and cisplatin changes when both the sequence and the time interval between the two treatments were varied in H28 cells. The cytotoxicity was not significantly enhanced by pre-treating the cells with either Mapatumumab or Lexatumumab, followed by subsequent cisplatin treatment, even when the time interval between the two treatments was increased up to 24 h (Fig. 5). Interestingly, increasing the time between cisplatin-treatment and subsequent treatment with either Mapatumumab or Lexatumumab did gradually increase the cytotoxic effects of both combinations over time in (Fig. 5).

Pre-treatment of H28 cells with cisplatin followed by treatment with either Mapatumumab or Lexatumumab resulted in 69% and 73% reduction in cell viability after 24 h for cisplatin with Mapatumumab or Lexatumumab, respectively (Fig. 5).

Thus, our findings highlight the importance of sequential administration of the drugs to increase the synergy.

### Cisplatin facilitates TRAIL receptor antibodies-mediated apoptosis

**Figure 6:**
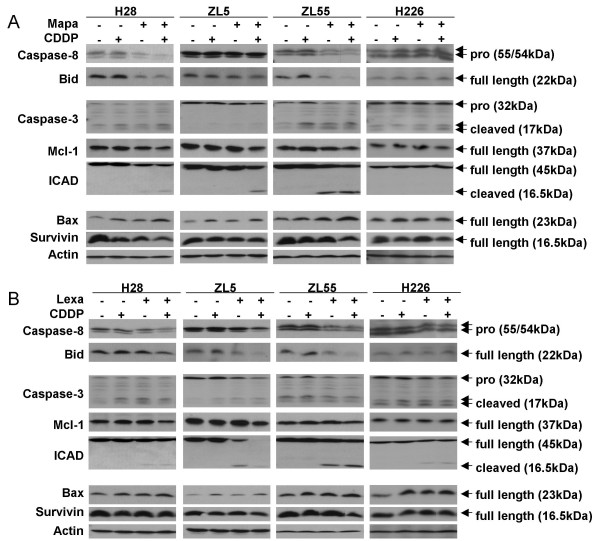
**Combined treatment with TRAIL receptor antibodies and cisplatin increases cell death**. H28, ZL5, ZL55 and H226 cells were cultured with or without 2 μM cisplatin for 24 h. Cells were then left untreated or treated with 10 μg/ml Mapatumumab (Mapa) ***(A) ***or Lexatumumab (Lexa) ***(B) ***for 24 h. Lysates were analyzed by immunoblotting as described under material and methods.

Mapatumumab, Lexatumumab and cisplatin are able to activate caspases [29-31]. To determine the molecular mechanisms involved in cisplatin enhancement of apoptosis induced by antibodies, four MPM cell lines with different sensitivities to Mapatumumab and/or Lexatumumab (H28, ZL5, ZL55 and H226, see Table 2) were treated with Mapatumumab or Lexatumumab in the presence or absence of cisplatin. Caspase 8, caspase 3 pathway and anti- or pro-apoptotic protein levels were measured to investigate the induction of apoptosis (Fig. 6A and Fig. 6B). Mapatumumab treatment led to cleavage of the TRAIL death effectors caspase 8 and Bid in H28 and ZL55 as would be expected based on results obtained by cytotoxicity assay. In ZL55 and to a lesser degree in H28 cells, Bid cleavage seems sufficient to trigger the intrinsic apoptosis pathway since activation of caspase 3 and cleavage of its substrate ICAD were observed (Fig. 6A). On the other hand, Lexatumumab, stimulated the TRAIL apoptotic pathway as expected in ZL5 and ZL55 (Fig. 6B). Cisplatin, which alone mainly increased BAX levels in all cell lines, in combination with Mapatumumab seemed to lower the threshold of extrinsic pathway stimulation since increased cleavage of TRAIL effectors was observed. A similar synergistic effect was observed by concurrent treatment with cisplatin and Lexatumumab in ZL5, and ZL55 cells compared to Lexatumumab alone. Little cleavage of TRAIL effectors was observed in the TRAIL resistant cell line H226 (Fig. 6A and Fig. 6B).

The most prominent increase in the processing of intrinsic pathway effectors caspase-3 and the caspase-3 targets ICAD and Mcl-1 were found in all tested cells lines after treatment with cisplatin in combination with either Mapatumumab or Lexatumumab (Fig. 6A and Fig. 6B).

Two recent studies, including ours, demonstrated that cisplatin-induced DNA damage up-regulates functional p53 in MPM [24,32]. Indeed, p53 negatively regulates the anti-apoptotic protein survivin in ZL34 MPM cell line [24,32]. In the four cell lines that we used in the present study, decreased survivin levels were observed only when a combination of cisplatin with Mapatumumab or Lexatumumab was used (Fig. 6A and 6B).

Taken together these results indicate that the synergism of combined cisplatin and agonist TRAIL receptor treatment is due to cross-talk between intrinsic and extrinsic apoptotic pathways.

### Cisplatin/TRAIL receptor antibodies-mediated cell death is inhibited by the antioxidant N-acetylcysteine

**Figure 7:**
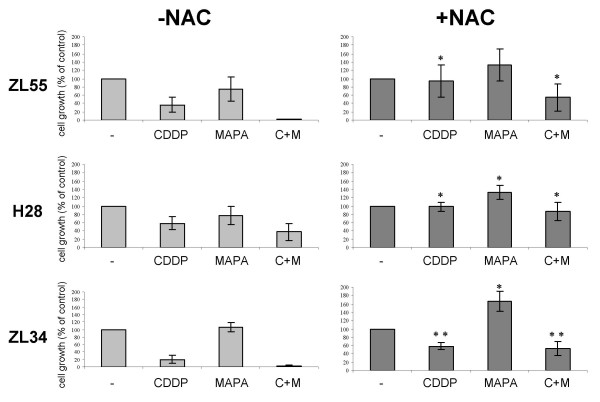
**The antioxidant NAC reduces cell death induced by either cisplatin, Mapatumumab or a combination thereof**. The MPM cell lines H28, ZL55 and ZL34 were treated with cisplatin (6.61 μM, 6.0 μM, and 6.0 uM, respectively) and 24 h later with Mapatumumab (0.13 μg/ml, 10 μg/ml, 10 μg/ml, respectively) as indicated, either in the presence or absence of NAC (5 mM). Cell proliferation was determined by MTT assay 48 h thereafter. Absorbance values obtained with untreated cells maintained under identical conditions were taken as 100%. Data represent means of at least three independent experiments and bars indicate standard deviations. **, *P *< 0.01 and *, *P *< 0.05 compared to cells treated in the absence of NAC but otherwise maintained under identical conditions.

It has been shown that cisplatin sensitization to the tumor necrosis factor receptor family member FasL is mediated by generation of oxidative stress [33]. In order to investigate whether the same mechanism was responsible for the observed synergism of combined cisplatin and agonist TRAIL receptor treatment we tested the effect of N-acetylcysteine (NAC), a well described scavenger of reactive oxygen species (ROS). NAC significantly reduced cell death by cisplatin and the combination treatment with cisplatin and Mapatumumab in ZL55, H28 and ZL34 cell lines (Fig. 7), indicating that the generation of ROS contributes to combination-induced cytotoxicity.

## Discussion

With TRAIL and TRAIL-receptor agonistic antibodies entering clinical trials for the treatment of patients with cancer [11], the question has arisen whether molecular markers can be identified, which would allow to select patients benefiting from such therapy.

Our results indicate that the majority of MPM cell lines (nine of thirteen) are sensitive to TRAIL. This is in contrast to previous studies reporting on a small number of cell lines only [19-22,34]. The potential advantage of targeting TRAIL pathway via agonistic antibodies for its receptors are the longer half-life compared to TRAIL and the higher specificity [23]. In agreement with a previous report [20], we have observed that MPM cells express membrane TRAIL-R1 and TRAIL-R2, with higher TRAIL-R2 expression. Studies based on receptor-blocking antibodies indicate that TRAIL can induce apoptosis through either TRAIL-R1 or -R2 or both receptors, but the relative contribution of each death receptor to apoptosis induction in cells expressing both receptors is unknown [10]. TRAIL-R2 was shown to contribute more that TRAIL-R1 to TRAIL-induced apoptosis in cells that express both death receptors [35]. However, a recent study [28] has shown that chronic lymphocytic leukaemia cells signal apoptosis exclusively via TRAIL-R1 despite surface expression of TRAIL-R2. Moreover, the apoptosis-inducing ability of different TRAIL preparations in various cells demonstrated an unanticipated preferential signalling via either TRAIL-R1 or -R2 [28]. We found that the majority of MPM cell lines were more sensitive to Lexatumumab (46%) than to Mapatumumab, while a minority (15%) were more sensitive to Mapatumumab than to Lexatumumab. The activity of both antibodies was similar in 2 cell lines (15%) and only few MPM cell lines were resistant to both (23%). Taken together, these results suggest that there is a permissive environment for preferential signalling via TRAIL-R2 to death-receptor mediated apoptosis in MPM cell lines expressing both receptors.

Comparison of TRAIL receptors expression levels and TRAIL sensitivity of the MPM cell lines used in this study did not reveal any consistent pattern, suggesting that TRAIL sensitivity is not dependent on TRAIL-receptor expression levels, thus indicating that other intracellular mechanisms control TRAIL signal transduction in resistant cells. TRAIL sensitivity can be regulated by anti-apoptotic proteins such as Bcl-2, Bcl-XL or FLIP [20]. We have shown in previous studies that Bcl-2 or Bcl-XL are abundantly expressed in MPM and it has already been demonstrated in MPM cells that downregulation of Bcl-XL is associated to sensitization to TRAIL apotosis [36]. This indicates that contrarily to what has been described in melanoma cell lines [37], expression level of TRAIL death receptors is not sufficient to identify MPM patients who may respond to TRAIL or to TRAIL-receptor agonistic antibodies.

No chemotherapy regimen for MPM has proven curative [5,6,38,39]. Therefore, new therapeutic options for the treatment of this malignancy need to be investigated. There is accumulating evidence indicating a synergism between anti-death receptor pathway and chemotherapy in the induction of apoptosis, although the synergistic mechanisms are not fully understood [40,41]. MPM cells have been found by others to be resistant to TRAIL-induced apoptosis, and require either chemotherapeutic drugs or cycloheximide to be combined with TRAIL for apoptosis to occur [19-21]. Our studies show that cisplatin synergistically enhances Mapatumumab- or Lexatumumab-mediated apoptosis in a caspase-dependent fashion and is also effective at promoting apoptosis when used in combination with either Mapatumumab or Lexatumumab in MPM tumor cells that are resistant to cisplatin, Mapatumumab or Lexatumumab single-agent therapy. We observed a high heterogeneity in the response of cell lines and primary cultures to treatment with TRAIL, Mapatumumab, Lexatumumab or cisplatin or a combination thereof. Cytotoxic chemotherapeutic drugs sensitize cultured cancer cells to TRAIL by different mechanisms including up-regulation of the receptors [42], enhanced death-inducing signalling complex formation or alteration of the expression of pro-apoptotic/anti-apoptotic proteins [31,43,44]. In previous studies, we showed that the expression of the anti-apoptotic proteins Bcl-XL and Bcl2 is highly variable in several MPM cell lines. This is consistent with data observed in a recent study using a large panel of MPM cell lines and tumors where highly variable expression levels of five inhibitor of apoptosis proteins, including survivin were found [45]. It is therefore likely that the observed differences in cell survival in cell lines and primary cultures upon treatment with cisplatin and TRAIL receptor agonistic antibodies are due to variation in basal expression levels of anti-apoptotic proteins.

The synergistic cytotoxicity between cisplatin and Mapatumumab or Lexatumumab is associated with an increase of caspase-mediated apoptosis. Indeed, the combination of cisplatin with Mapatumumab or Lexatumumab synergistically enhanced caspase-8 and Bid activation in MPM cells sensitive to antibody treatment and caspase-3 activation in all cells treated with a combination of cisplatin and Mapatumumab or Lexatumumab. In a previous study we have demonstrated that p53 is functional in MPM cells and that it negatively regulates the anti-apoptotic protein survivin [24,32]. Combination of cisplatin with Mapatumumab or Lexatumumab further increased the expression of p53 transcriptional targets Bax and decreased survivin, compared to the treatment with either agent alone. Similarly, decrease in anti-apoptotic Mcl-1 expression was observed upon exposing MPM cells to the combination of cisplatin with Mapatumumab or Lexatumumab, confirming the significant role of these proteins in the enhancement of death receptor-mediated apoptosis by cisplatin in MPM cells. Based on the scavenging effect of the antioxidant antioxidant NAC on cell death induced either by cisplatin and the combination of Mapatumumab and cisplatin, we infer that the molecular mechanism responsible for the synergism is linked to the production of reactive oxygen species, which act as positive regulator of apoptosis. The role of cisplatin-induced oxidative stress in the enhancement of the efficiency of TNF family members has already been described in MPM cell lines after treatment with cisplatin, FasL or the combination thereof [33]. Our findings are also in agreement with a study showing that the generation of ROS sensitizes colon cancer cells to death-inducing ligand TRAIL [46] and with a study showing that ROS generation by Sulforaphane is pivotal for the sensitization of hepatoma cells to TRAIL-induced apoptosis [47].

Contrary to what has been observed in squamous cell carcinoma and the ligand TRAIL [31], we have observed that pre-treatment with cisplatin followed by treatment with Mapatumumab or Lexatumumab resulted in significantly higher cytotoxic effects in MPM cell lines than when the sequence was reversed. The reasons for such a difference are not clear and can include different kinetics of apoptotic pathways in different cell lines [48] and/or dosage.

## Conclusion

In summary, our results indicate that the sequential administration of cisplatin followed by the human agonistic TRAIL receptor antibodies Mapatumumab or Lexatumumab deserve investigation in the treatment of patients with MPM.

## Methods

### Cell culture and reagents

The MPM cell lines SCP212, ZL34,, SPC111, ZL5, ZL55 and the primary cultures SDM4 SDM6 and SDM13 were generated in our laboratory and have been described previously [24,25]. The MPM cell lines H2052, H226, H2452, H28 and MST0-211H were obtained from ATCC (LGC Promochem Sarl, France). All cells were maintained in RPMI 1640 (Sigma, St. Louis, MO, USA) supplemented with 2 mM L-glutamine, 1 mM sodium pyruvate, 10% FBS and 1% (w/v) penicillin/streptomycin. Jurkat cells were obtained from ATCC and maintained in RPMI 1640 medium (Sigma) supplemented with 5% FBS (Invitrogen, Paisley, UK), 15 mM HEPES, 2 mM L-glutamine, 50 μM β-mercaptoethanol and 1% (w/v) penicillin/streptomycin (Invitrogen). All cells were grown at 37°C in a humidified atmosphere containing 5% CO_2_.

### Reagents

Recombinant human polyhistidine-tagged TRAIL (His-TRAIL) and antibodies to TRAIL-R1, TRAIL-R2, TRAIL-R3 and TRAIL-R4 were purchased from Alexis (Lausen, Switzerland). The agonistic monoclonal antibodies against TRAIL-R1 (Mapatumumab) and TRAIL-R2 (Lexatumumab) were provided by Human Genome Sciences (Rockville, MD, USA). The following antibodies were purchased: caspase-8 (Alexis, Lausen, Switzerland), Bid (R&D Systems, Minneapolis, MN, USA), Mcl-1 (Santa Cruz Biotechnology, Santa Cruz, CA, USA), ICAD (Santa Cruz Biotechnology, Santa Cruz, CA, USA), caspase-3 (BD PharMingen, San Diego, CA, USA), Bax (Santa Cruz Biotechnology, Santa Cruz, CA, USA), survivin (R&D Systems, Minneapolis, MN, USA), and actin (ICN Biomedicals, Irvine, CA, USA). Where indicated, either cisplatin (Bristol-Myers Squibb AG, Baar, Switzerland) and/or N-acetyl-L-cysteine (ALEXIS Corporation, Lausen, Switzerland) were added.

### Measurement of cell growth

Cell growth was determined using colorimetric cell viability assay based on the reduction of tetrazolium salt MTT, as described [49]. Cells were plated in quadruplicate in 96-well plates (7500 cells/well) and absorbance was measured using a SPECTRAmax 340 microplate reader. Cell growth was calculated as a percentage of the absorbance signal obtained with wells of untreated (viable) cells kept under identical conditions. Dose curve plots, IC50 and Combination Index (CI) were calculated by using CalcuSyn software from BIOSOFT.

### Immunoblotting

Cells were lysed for 30 min with 1× RIPA buffer (Upstate) containing 0.1% SDS, 1 mM PMSF and complete protease inhibitor cocktail (ROCHE). Lysates were clarified by centrifugation (10,000*g *for 30 min at 4°C) and protein concentrations were determined using BCA (Pierce/Perbio Science S.A., Lausanne, Switzerland). After SDS/PAGE separation, the protein was transferred to nitrocellulose membrane and immunoblotting was performed as described [50] using the specific antibodies mentioned above.

### Flow cytometric analysis

Immunostaining of intact cells was performed as described previously. Surface expression of TRAIL receptors was evaluated by indirect immunostaining using the primary antibodies mentioned above followed by PE-conjugated anti-mouse secondary antibodies (Alexis Biochemicals). Nonspecific fluorescence was assessed using normal mouse immunoglobulin G (IgG) followed by secondary antibodies. Flow cytometric analyses were performed using a FACSCalibur (FACScan, BD Biosciences, San Jose, CA, USA).

### Statistical analysis

Data are presented as the mean ± SE of at least three independent experiments. Statistical differences were assessed using two-sided unpaired Student's *t *test and P values < 0.05 were considered significant.

## Competing interests

The author(s) declare that they have no competing interests.

## Authors' contributions

LLB and TMM carried out the molecular biology studies and participated in the interpretation of the data and drafted the manuscript. SK carried out the survival- and immunoassays. SHD and EFB were involved in drafting the manuscript and revising it critically for important intellectual content. RAS was involved in drafting the manuscript, revising it critically for important intellectual content and has given final approval of the version to be published. All authors read and approved the final manuscript.
